# Predictors of exercise participation in ambulatory and non-ambulatory older people with multiple sclerosis

**DOI:** 10.7717/peerj.1158

**Published:** 2015-08-06

**Authors:** Michelle Ploughman, Chelsea Harris, Elizabeth M. Wallack, Olivia Drodge, Serge Beaulieu, Nancy Mayo, Health Lifestyle and Aging with MS Canadian Consortium

**Affiliations:** 1 Recovery and Performance Laboratory, Rehabilitation Research Unit, Faculty of Medicine, Memorial University, St. John’s, NL, Canada; 2 Eastern Health Authority, St. John’s, NL, Canada; 3 Department of Clinical Epidemiology, McGill University, Montreal, QC, Canada

**Keywords:** Disability, Physical activity, Rehabilitation, Exercise behavior, Physiotherapy, Progressive multiple sclerosis, Perseverence, Resilience, Self-efficacy, Depression

## Abstract

**Background.** Exercise at moderate intensity may confer neuroprotective benefits in multiple sclerosis (MS), however it has been reported that people with MS (PwMS) exercise less than national guideline recommendations. We aimed to determine predictors of moderate to vigorous exercise among a sample of older Canadians with MS who were divided into ambulatory (less disabled) and non-ambulatory (more disabled) groups.

**Methods.** We analysed data collected as part of a national survey of health, lifestyle and aging with MS. Participants (*n* = 743) were Canadians over 55 years of age with MS for 20 or more years. We identified ‘a priori’ variables (demographic, personal, socioeconomic, physical health, exercise history and health care support) that may predict exercise at moderate to vigorous intensity (>6.75 metabolic equivalent hours/week). Predictive variables were entered into stepwise logistic regression until best fit was achieved.

**Results.** There was no difference in explanatory models between ambulatory and non-ambulatory groups. The model predicting exercise included the ability to walk independently (OR 1.90, 95% CI [1.24–2.91]); low disability (OR 1.50, 95% CI [1.34–1.68] for each 10 point difference in Barthel Index score), perseverance (OR 1.17, 95% CI [1.08–1.26] for each additional point on the scale of 0–14), less fatigue (OR 2.01, 95% CI [1.32–3.07] for those in the lowest quartile), fewer years since MS diagnosis (OR 1.58, 95% CI [1.11–2.23] below the median of 23 years) and fewer cardiovascular comorbidities (OR 1.55 95% CI [1.02–2.35] one or no comorbidities). It was also notable that the factors, age, gender, social support, health care support and financial status were not predictive of exercise.

**Conclusions.** This is the first examination of exercise and exercise predictors among older, more disabled PwMS. Disability is a major predictor of exercise participation (at moderate to vigorous levels) in both ambulatory and non-ambulatory groups suggesting that more exercise options must be developed for people with greater disability. Perseverance, fatigue, and cardiovascular comorbidities are predictors that are modifiable and potential targets for exercise adherence interventions.

## Introduction

Exercise training has the potential to mitigate the symptoms of multiple sclerosis (MS), a neurological disease characterized by unpredictable progressive episodes of inflammation and demyelination of the central nervous system (Latimer-Cheung et al., 2013; Prakash et al., 2010). The potential role of exercise to slow MS progression, preserve neuronal integrity and promote healthy aging is gaining interest (Dalgas & Stenager, 2012), however, people with MS engage in lower levels of exercise when compared to the general population (Motl & McAuley, 2009; Stroud, Minahan & Sabapathy, 2009; Van der Ploeg et al., 2007). With high exercise drop-out rates (Kayes et al., 2011; Ploughman et al., 2012a), nearly 80% of relapsing-remitting MS patients are not reaching the public health recommended guidelines of moderate-to-vigorous physical activity (Klaren et al., 2013). Understanding the factors predicting exercise participation among people with MS-related disability is the first step in developing new strategies to promote exercise.

Most studies examining predictors of exercise in MS do not distinguish between physical activity and exercise (Boslaugh & Andresen, 2006; Dalgas & Stenager, 2012). Although the terms are sometimes used interchangeably, they are different in that physical activity is any activity that is part of everyday life, while exercise is planned and structured intended to improve or maintain physical fitness (ACSM, 2010). As emerging evidence suggests that exercise at moderate to high intensity (as opposed to light physical activity) is neuroprotective (Austin et al., 2014; Klaren et al., 2014; Ploughman et al., 2015), predictors specific to higher intensity training may be important for exercise prescription. Previous studies have reported that exercise barriers in MS are primarily level of disability, fatigue (Asano et al., 2013) and self-efficacy (Stroud, Minahan & Sabapathy, 2009) while physical activity predictors are level of disability, enjoyment, and social support (Motl et al., 2006). In one study of moderate to vigorous exercise activity predictors among people with spinal cord injury (SCI; mean age 47 and average 15 years post-injury), strongest exercise predictors were positive exercise intentions and number of years post-injury. Greater social integration, physical independence and employment were also associated with exercise (Ginis et al., 2012). Whether these factors also apply to people with MS-related disability is not known. Considering the importance of exercise, understanding the barriers to participation in exercise at intensities high enough to induce a training effect is imperative for future MS clinical trials.

Almost all studies in MS and exercise recruit subjects at the early phase of the disease (Rietberg et al., 2005). Older people with MS are often excluded from MS research (Ploughman et al., 2012a; Ploughman et al., 2012b; Ploughman et al., 2014). Several authors in systematic reviews have expressed an urgent need to examine exercise interventions among people with more advanced MS-related disability (Latimer-Cheung et al., 2013; Rietberg et al., 2005). In this study we aimed to determine the factors predicting exercise adherence (at American College of Sports Medicine (ACSM) recommended levels) among older people with MS in order to design more tailored interventions across disability levels. We hypothesize that predictors of exercise will be different between people with MS who are ambulatory and those who are non-ambulatory.

## Methods

### Survey design

We accessed and performed secondary analysis of data collected from 743 people with MS as part of a national survey; the ‘Canadian Survey of Health, Lifestyle and Aging with MS’ (Ploughman et al., 2014) which was approved by 11 health research ethics boards across Canada. The database included health and lifestyle variables obtained from self-report questionnaires mailed to participants over the age of 55 years with MS symptoms for more than 20 years. Complete survey methods are described elsewhere (Ploughman et al., 2014).

### Potential predictive variables

Based on previous research (Asano et al., 2013; Kayes et al., 2011; Motl et al., 2006; Stroud, Minahan & Sabapathy, 2009) and a model of healthy aging created using a qualitative approach (Ploughman et al., 2010; Ploughman et al., 2012a; Ploughman et al., 2012b; Ploughman et al., 2014) a list of ‘a priori’ factors potentially associated with exercise adherence were categorized into six domains; (1) demographic, (2) personal, (3) socioeconomic, (4) physical health, (5) exercise history and (6) healthcare support. Demographic information included age, gender, years of education, type of MS at diagnosis and years since MS diagnosis. Personal factors included stress, measured as part of the Simple Lifestyle Indicator Questionnaire (SLIQ) (Godwin et al., 2008), mood, measured using the Hospital Anxiety and Depression Scale (HADS) (Zigmond & Snaith, 1983), and resilience (the Resilience Scale) (Wagnild, 2009). HADS and the Resilience Scale were separated into their subcomponents; HADS into anxiety (HADS-A) and depression (HADS-D) and resilience into five aspects (equanimity, perseverance, self-reliance, meaningfulness, and existential aloneness) (Wagnild, 2009).

Physical health variables included disability, measured by the Barthel Index (Mahoney & Barthel, 1965), fatigue (rated with a visual analogue scale), and co-morbid conditions determined using the Co-morbidity Questionnaire developed by Marie and Horwitz (Marrie & Horwitz, 2010). Socioeconomic variables included financial situation and social support. Level of social support was measured using the Personal Resource Questionnaire-2000 which consists of 15 items with a score range from a low of 7 to high of 105 (Weinert & Brandt, 1987). To determine health care support, participants identified and ranked the helpfulness of health care providers on a scale of 1 (not helpful) to 5 (very helpful). Participation in exercise and other lifestyle habits (smoking, alcohol, diet) was collected from responses to the Simple Lifestyle Indicator Questionnaire (SLIQ) (Godwin et al., 2008). In addition to describing their current level of exercise, respondents were also asked to describe the type and intensity of exercise they had engaged between the ages of 20 and 30 years (past exercise experience).

All the survey measures (and the survey itself) had been tested or validated among people with MS or community-living older adults. Complete information is provided elsewhere (Ploughman et al., 2014). The Barthel Index, SLIQ and Personal Resource Questionnaire 2000 were pretested using cognitive debriefing (Ploughman et al., 2010).

### Data analysis

The statistical methods follow the steps outlined in Table 1. To create the outcome, descriptive physical activity information from SLIQ was transformed into metabolic equivalents (METS; a measure that quantifies exercise intensity based on the ACSM guidelines (ACSM, 2010)), and then MET-hours per week (MET intensity × number of 1 h intervals of exercise per week). As this measure was highly skewed, we classified respondents into ‘Exercisers’ (>6.75 MET-hours per week) or ‘Non-exercisers’ (<6.75 MET hours per week). These cut-off values were based on the ACSM recommendation that in order to improve or maintain fitness people with MS should be active three times weekly for 20–30 min at a moderate intensity (∼4.5 METs × 3 × 30 min sessions = 6.75 MET-hours/week) (ACSM, 2010).

**Table 1:** Statistical methods.

Step #	Description
1	Examine the distribution of the outcome variable. If it is too skewed to model with linear regression, create a binary variable using theoretical considerations when available, or otherwise based on the statistical distribution.
2	Test univariate models with each predictor. If the predictor is ordinal or continuous, test two models: one with the predictor as continuous, the other as categorical, based either on variable categories or on quartiles. Examine estimates and AIC of each model. If the model with categorical estimates results in an AIC closer to 0, retain the ordinal format for that variable. If the categorical format is retained but four levels are not needed, reduce the variable to a binary format.
3	Test models of each predictor with ambulatory status and an interaction term to identify any predictors that have different associations with the outcome for ambulators and persons with limited ambulation.
4	Test an initial multivariate model of all predictors and interactions with *p* < 0.10.
5	Remove one by one, predictors with *p* values >0.05, starting with the predictor with the highest *p*-value (for the interactions, base decision on the *p*-value of the interaction term).
6	Once a model of only significant terms is obtained, add back each excluded variable, one by one.
7	Test a new multivariate model that includes any new variables with *p* > 0.10 identified in Step 6.
8	Redo Step 5 until a final model is obtained except retain variables that improve fit even if they don’t attain significance at *p* < 0.05

In the first step of building an explanatory model, each ‘a priori’ variable (independent variable) was separately entered into a simple binary logistic regression with the dependent variable (exercise, no exercise). Continuous variables were modelled both linearly and as categories based on 25th, 50th and 75th quartiles or based on accepted cut-offs from the literature. Linear fit was examined by comparing Aikaike Information statistics (AIC) over models. When the AIC of the model with categorical variables was better than that with the continuous variable, that variable was retained only in the categorical format. When four levels were not required to categorize the variable, levels were combined and the variable retained in a binary format.

It was our hypothesis that predictors of exercise would differ among people with different degrees of MS disability. A variable indicating ambulation was therefore created from the response to the ambulation question in the Barthel Index. Those who scored 0 or 5 (answers walk independently with or without a cane >150 m) were categorized as ‘Ambulators’. Respondents who scored 10 or 15 on Barthel Index ambulatory question (answers use of wheelchair/walking aid indoors only) were categorized as ‘Non-Ambulators’. All predictor variables were tested for interaction with ambulation (step 3; Table 1): a main effect of and an interaction with ambulatory status were added to each of the univariate models to determine if the association with exercising was different for persons who were ambulatory and those who were not.

In step 4, all variables (linear or categorical, with or without an associated interaction) that predicted exercise, (*p* < 0.10) were entered into a multivariate logistic regression model. Variables with p-values above 0.05 were removed from the model one by one, based on their p-values (step 5; Table 1). Once all remaining variables were statistically significant, all variables that had been removed were re-entered one by one as well as with an interaction for ambulation to verify that none was eligible to re-enter the model (step 6; Table 1).

Because it was hypothesized, ‘a priori’, that predictors of exercising would vary between ambulators and non-ambulators, an internal cross validation (Kleinbaum, Kupper & Muller, 1988) was also done to examine whether a model created among ambulators would also fit non-ambulators. To do this, betas from a logistic regression model created among ambulators was applied to non-ambulators and *c*-statistics of the two compared.

Differences between exercisers and non-exercisers were compared using ANOVA and chi-square; significance set at *p* < 0.05. Statistical analyses were performed using SAS 9.3.

## Results

### Participant characteristics

Respondents were on average 64.6 (SD6.2) years of age and had been diagnosed with MS on average 24.8 (SD10.0) years prior. The ratio of females to males was 3.5:1. In comparing the characteristics of Exercisers (*n* = 404) and Non-exercisers (*n* = 339), the Non-exercisers were older and more disabled (as measured by the Barthel Index) (Table 2). They were more likely to be initially diagnosed with primary or secondary progressive disease and less likely to be diagnosed with relapsing-remitting MS (Table 2). When describing their past exercise experience, 368/743 (50%) respondents reported that they were previously active but are now inactive. One hundred and four respondents (14%) reported being inactive for most of their life (not active during the ages 20–30 and not currently active) and 271 (36.5%) reported that they were active when they were young and are still currently active. Those who described their current exercise reported participating in activities such as swimming and water fitness (*n* = 82), gardening and housework (*n* = 114), yoga, stretching or Tai Chi (*n* = 84) and most commonly, walking (*n* = 266).

**Table 2:** Characteristics of exercisers and non-exercisers.

Self-reported characteristics		Exerciser *N* = 404 mean (±SD)	Non-exerciser *N* = 339 mean (±SD)
Age		64.1 (±5.8)	65.1 (±6.5)^*^
Total education		13.6 (±2.5)	13.3 (±2.7)
Gender	Male	83	83
	Female	321	256
Years since diagnosis		23.6 (±10.2)	26.3 (±9.7)^**^
Barthel Index		87.0 (±14.7)	63.2 (±28.0)^**^
Type of MS (Initial diagnosis)	Relapsing-Remitting	234 (60.6%)	152 (39.4%)^*^
	Primary progressive	40 (37.5%)	59 (62.5%)^*^
	Secondary progressive	26 (40.0%)	39 (60.0%)^*^
	Progressive relapsing	7 (43.8%)	9 (56.3%)
	Benign	26 (60.5%)	17 (39.5%)
	Unknown	68 (54.0%)	58 (46.0%)

**Notes.**

* *p* < 0.05.

** *p* < 0.01.

### Predictors of exercise

Of the independent variables considered, many had a significant univariate association with exercising, though only meaningfulness (part of the Resilience Scale), fatigue and financial situation had associations that varied by ambulatory status (*p* < 0.10).There were expected correlations between predictor variables (>0.3); resilience, depressive symptoms, anxiety symptoms, fatigue and social support (data not shown). A final predictive model was created (Table 3). Ambulators had a greater odds of exercising at moderate to vigorous levels (OR 1.90, 95% CI [1.24–2.91]), as did people with lower levels of disability generally (OR 1.50, 95% CI [1.34–1.68] for each 10-point difference in the Barthel Index). Similarly, the odds of exercise were higher among persons with higher levels of perseverance.17, 95% CI [1.08–1.26] for each additional point on the scale of 0 to 14), less fatigue (OR 2.01, 95% CI [1.32–3.07] for those in the lowest quartile), fewer years since diagnosis of MS (OR 1.58, 95% CI [1.11–2.23] for those diagnosed within the past 23 years compared to 24 years ago or more), and less cardiovascular comorbidity (OR 1.55, 95% CI [1.02–2.35] for no more than one compared to two or more). As perseverance has a standard deviation of 2.3, a 1-point difference on this scale is close to a clinically significant difference based on a half a standard deviation. For easier interpretation, compared to the worst quartile of perseverance (scores from 2 to 9), odds ratios for the second (10–11), third (12) and best (13–14) quartiles were 1.50 (95% CI [0.89–2.51]), 2.04 (95% CI [1.19–3.49]) and 2.71 (95% CI [1.62–4.48]) respectively. Similarly, modelling the Barthel Index as quartiles compared to the worst quartile (scores of 0–60), results in odds ratios of 4.11 (95% CI [2.39–7.06]), 7.04 (95% CI [3.91–12.68]) and 10.1 (95% CI [5.06–20.28]) for the second (scores 65–80), third (scores 85–90) and best (a score of 100) quartiles, respectively. This model had good fit based on AIC statistics of other models considered. Furthermore, the *c*-statistic of 0.81 is considered strong (Hosmer & Lemeshow, 1989; Hosmer & Lemeshow, 2000): a *c*-statistic of 0.5 indicates allocation no better than chance and a value of 1.0 perfect assignment.

**Table 3:** Explanatory model of exercise participation.

Model components	Description	*β*	Odds ratio: exp(*β*)	95% CI of exp(*β*)
Ambulatory status	Non-Ambulatory		Reference	
	Ambulatory	0.64^**^	1.90	1.24, 2.91
Barthel Index (disability) score	10 points less disability on a scale of 0–100	0.41^***^	1.50	1.34, 1.68
Perseverance	1 point more perseverance on a 0–14 scale	0.16^***^	1.17	1.08, 1.26
Fatigue today	3 lowest quartiles on a scale of 0–100		Reference	
	Best quartile	0.70^**^	2.01	1.32, 3.07
Years since MS diagnosis	Above the median (24–66 years)		Reference	
	Below the median (0–23 years)	0.45^*^	1.58	1.11, 2.23
Cardiovascular comorbidity	2 or more		Reference	
	None or 1	0.44^*^	1.55	1.02, 2.35

**Notes.**

* *p* < 0.05.

** *p* < 0.01.

*** *p* < 0.001.

Exp (*β*): exponential of the *β* coefficient yields the OR.

To determine if separate statistical models were needed to predict exercising among ambulatory and non-ambulatory persons, internal cross-validation methodology was performed by fitting a logistic regression model to ambulators, applying the regression coefficients for the explanatory variables (*β*) to the observed data on these variables among the non-ambulators, and comparing fit via c-statistics (Kleinbaum, Kupper & Muller, 1988). For ambulators alone, fatigue was replaced with a measure of depressive symptoms. Fatigue and depressive symptoms were correlated, but while fatigue was a better fit for the model of ambulators and non-ambulators combined, depression improved fit among ambulators only. The *c*-statistics among ambulators was 0.71 (95% CI [0.65–0.78]), and that of non-ambulators 0.74, supporting the determination that only a single model was needed to determine predictors of exercise.

## Discussion

To our knowledge this is the first examination of exercise predictors in a sample of people with a full range of MS-related disability; from independently ambulatory to completely dependent for activities of daily living (ADL). We divided the sample of 743 older Canadians with MS into ambulatory (low disability) and non-ambulatory (higher disability and more progressive disease) groups in order to determine if the predictors would differ between the groups; critical knowledge in order to promote exercise compliance in future MS clinical exercise trials. We also applied strict criteria to delineate exercise levels based on ACSM guidelines as a growing body of research suggests that moderate exercise (not light physical activity) may be neuroprotective (Austin et al., 2014; Dalgas & Stenager, 2012). We found that predictors of exercise were the same for older people with MS whether they were ambulatory or not. Ambulatory status, disability, perseverance, fatigue, years since MS diagnosis and cardiorespiratory comorbidity explained exercise participation among older people with MS.

### Level of disability a major predictor

Level of disability was the major predictor of exercise in both ambulatory and non-ambulatory groups. Respondents scoring 100/100 on Barthel Index (highest quartile; least disability) were 10 times more likely and those scoring 85–90, seven times more likely to exercise than those scoring less than 60 (lowest quartile). Ambulatory people with MS were 1.9 times more likely to exercise than those who were non-ambulatory (walked indoors only, used a wheelchair or were bedridden). Furthermore, respondents diagnosed for less than 24 years were 1.6 times more likely to exercise than those diagnosed longer than 24 years. Taken together, our results suggest that respondents who need assistance or who have walking disability have few exercise options. Development of seated and modified exercise programs followed by effectiveness research is required. Programs that are individually tailored, guided by qualified personnel such as a physiotherapist, focused on goal-setting and independence using remote technology such as Blue Prescription, hold promise (Hale et al., 2013). Several systematic reviews examining exercise in MS suggest a critical need for rehabilitation research among more disabled groups (Latimer-Cheung et al., 2013; Rietberg et al., 2005). Although our sample were on average 64 years of age with MS symptoms for about 33 years, our finding of the critical role of disability in exercise concurs with findings from a sample of 417 ambulatory participants who were on average 43 years old with symptoms for about 8 years (Asano et al., 2013) and among 68 people with relapsing-remitting MS (Suh et al., 2014).

Our findings also suggest that the predictors of exercise participation in MS differ somewhat from those of people with SCI (Ginis et al., 2012). Martin Ginis and group showed that physical independence and injury severity were not strongly predictive of exercise participation in middle-aged people with chronic spinal cord injury (Ginis et al., 2012), suggesting that interventions to promote exercise compliance in SCI may not be entirely applicable to MS.

### Perseverance

A novel finding in this study was the role of resilience, specifically perseverance, in predicting exercise participation. Respondents scoring in the highest quartile of perseverance (scores of 13 or 14 out of 14) were 2.7 times more likely to exercise than those scoring in the lowest quartile (less than 10). In our previous qualitative research (Ploughman et al., 2012a; Ploughman et al., 2012b), perseverance, or commitment to an outcome despite challenges, had previously emerged as a characteristic of people who maintained exercise into old age despite their MS-related symptoms. Previous results of the ‘Canadian Survey of Health Lifestyle and Aging with MS’ database showed that this sample of older people with MS exercise more than other older Canadians (Ploughman et al., 2014).

Other studies have shown that self-efficacy (Kasser & Kosma, 2012; Kosma & Kasser, 2012; Nickel et al., 2014; Schmitt et al., 2014), perceived exercise benefits (Kosma & Kasser, 2012; Suh et al., 2014) and positive exercise intentions (Ginis et al., 2012) are associated with exercise participation in people with MS and SCI-related mobility disability. Self-efficacy, resilience and perseverance are overlapping constructs that impact health behaviors (Pilutti et al., 2014; Sinnakaruppan et al., 2010). Resilience is believed to be an innate characteristic but research suggests that it can also be learned (McAllister & McKinnon, 2009). Researchers have used focused cognitive behavioural techniques to encourage optimism and dispute pessimistic thinking as a method to improve individual resilience and self-efficacy (Graziano et al., 2014). Resilience can be improved using optimism training, as well as teaching control and empowerment, educating individuals about their illness, and involving patients in support groups (Ng et al., 2013). These techniques are clearly important in promoting exercise participation and should be considered in the design of exercise trials especially among people with significant MS-related barriers.

### Fatigue or depression as predictors?

We found that participants who reported less fatigue (top quartile in 0-100 scale) were twice as likely to exercise. Asano and group (Asano et al., 2013) have also reported fatigue (feeling too tired) as an exercise barrier among people with MS. It is important to consider that MS fatigue and depressive symptoms overlap and in fact, fatigue is an attribute of depression suggesting a complex relationship between the two (Feinstein et al., 2014). Presence of depressive symptoms provided a slightly better model fit than fatigue in the ambulatory group during our analysis. Motl et al. also showed that depression was a symptom inversely associated with exercise in a group of ambulatory subjects with relapsing–remitting MS (Suh, Motl & Mohr, 2010). Whether fatigue or depression or both, better symptom management is required in order to foster exercise participation in the long term.

### Comorbid conditions

As our study cohort were older, we expected participants to have more comorbid conditions, however, to our knowledge this is the first report that the number of cardiovascular comorbid conditions impacted exercise behaviour in MS. It was interesting that cardiovascular conditions (diabetes, hypertension and hyperlipidemia) but not musculoskeletal conditions (arthritis, joint replacement etc.) predicted exercise. Participants reporting fewer than two cardiovascular comorbidities were 1.5 times more likely to exercise than those reporting more. As reported by Marrie and Hanwell, comorbid conditions are associated with increased MS disability progression and lower adherence to treatment which could be linked to low exercise participation (Marrie & Hanwell, 2013). Exercise participation, adherence to a healthy diet and avoidance of smoking and excessive alcohol are likely parts of an overall MS self-management program to enhance healthy aging with MS (Ploughman et al., 2012a; Ploughman et al., 2012b; Ploughman et al., 2014).

### Factors that do not predict exercise

Previous research suggests that as individuals age they experience a progressive loss of cognitive and physical skills and abilities, which act as barriers for engagement in healthy lifestyle practices, like physical activity (Widerstrom-Noga & Finlayson, 2010; Motl et al., 2006; Prakash et al., 2010). Our findings did not support age as an exercise predictor.

Based on previous qualitative and quantitative we had expected that gender (Anens et al., 2014), social support (Ploughman et al., 2012a), financial resources (Ginis et al., 2012), previous exercise behaviors and the support of health care professionals (Ploughman et al., 2012b) would be predictive of exercise participation but they were not. When subjected to rigorous analysis in a large cohort with MS-related disability, the influence of these factors were negligible and even absent. On the other hand, our cohort of older people with MS was unique so the impact of gender as reported by others (Anens et al., 2014) and other differences may not be as applicable in this group.

### Limitations

Although this unique cohort may provide new insights into maintaining exercise participation among people with MS as they age, there are some study limitations. The cross-sectional design limits our ability to assess change and the effects of variables on predicting exercise participation over time. As the data analysed was obtained through surveys, the responses to the questions were subjective which may or may not align with objective measures. Furthermore, questions such as past exercise experience and diagnosis of co-morbid conditions can be affected by recall bias and memory difficulties. By nature of the volunteer survey design, our sample may be biased in that active participants and those without cognitive impairment may have been more likely to respond. We did not examine cognitive impairment, nor did we have access to data about sleep patterns and pain; potential moderators of exercise and physical activity.

## Conclusion

This study sought to determine the factors predicting exercise adherence among older people with MS-related disability. We found level of disability and perseverance to be strong predictors whether participants were ambulatory or not. Clearly, in order for older people with MS to maintain exercise participation as they age they need exercise tailored to their abilities paired with techniques to overcome challenges that arise. Our findings also show that fatigue and cardiovascular comorbid conditions are modifiable barriers to exercise.

## Additional Information and Declarations

### Data Availability

The following information was supplied regarding the deposition of related data: Dr. Michelle Ploughman is the data custodian for the data contained within the Health Lifestyle and Aging with MS Survey. The data is maintained in a secure network as per ethics requirements. Access to the data can be requested by contacting Dr. Ploughman at michelle.ploughman@med.mun.ca.
